# Proceedings of the International Hahnemannian Association

**Published:** 1890-09

**Authors:** J. W. Thomson

**Affiliations:** 114 West Sixteenth Street, New York


### PROCEEDINGS OF THE INTERNATIONAL HAHNEMANNIAN ASSOCIATION.

114 West Sixteenth Street,
New York, July 22d, 1890.

To the Editors of The Homœopathic Physician :—
Referring to the publication of the Proceedings of the International Hahnemannian Association, every member ought surely to have a copy just as soon as possible to get them into print. They would also conserve the highest possible use. The members cannot have the Proceedings too soon to study and be enriched and blessed by them. Notwithstanding the earnest and indefatigable way in which the sessions of the Association were conducted, probably less than half of the papers were read. And we want the whole of this rich treasury before us as soon as may be. And to insure accuracy we ought to have the Proceedings published under the ægis of the Society. It really seems all wrong to let any of these valuable papers pass from the possession of the Society until they are published intact, with the discussions that followed. Those of the members who were not present may thus obtain some faint perception of the rich *aura* of the meeting that blessed those who were at Watch Hill.

This waiting nearly a year is all wrong. I only obtained a copy of last year's transactions a few days before the last meeting, and then only after I had made special application to the Secretary.

Pray let us have the transactions of 1890 as soon as they can be published.

J. W. Thomson.

